# The Search for Biochemical Markers

**Published:** 1995

**Authors:** Robert M. Anthenelli, Boris Tabakoff

**Affiliations:** Robert M. Anthenelli, M.D., is an assistant professor of psychiatry at the University of California, San Diego, School of Medicine, and director of the Dual Diagnosis Treatment Program, San Diego Veterans Affairs Medical Center, San Diego, California. Boris Tabakoff, Ph.D., is a professor and chair of the Department of Pharmacology, University of Colorado Health Sciences Center, Denver, Colorado

**Keywords:** AOD dependence, biological marker of potential AODD (alcohol and other drug use disorders), genetic trait, platelets, monoamine oxidase, adenylate cyclase, enzyme test, hereditary factors, risk factors

## Abstract

Scientists are searching for biochemical markers for alcoholism that would allow researchers to distinguish easily and reliably between alcoholics and nonalcoholics or between people at risk for alcoholism and those not at risk. Two proposed markers that have been studied intensively are the activity levels of the enzymes platelet monoamine oxidase (MAO) and platelet adenylyl cyclase (AC). Levels of MAO activity, which are genetically determined, often are significantly lower in alcoholics than in nonalcoholics. Recent studies indicate that low MAO activity levels may be a marker for a subtype of alcoholism that is characterized by an earlier age at onset. AC activity levels also are genetically determined and frequently reduced in alcoholics, compared with nonalcoholics, even after alcoholics experience long periods of abstinence. Thus, AC activity levels also may be a marker for alcoholism, although research findings indicate that low AC activity may be characteristic of a different alcoholism subtype than low MAO activity. Further studies will analyze in more detail the significance of MAO and AC activity levels as markers for alcoholism and investigate their potential role in the diagnosis, disease process, and treatment of alcoholism.

An important focus of alcohol research is the search for biochemical characteristics, or markers, that could distinguish people who are alcoholic or who have a predisposition for alcoholism from people who do not. Because such markers would represent inborn characteristics, or traits, they also are called trait markers. The identification of reliable trait markers could potentially reduce the damage caused by alcoholism. For instance, trait markers could provide the basis of screening tests for use in high-risk populations (e.g., children of alcoholics) to allow early identification of persons who are vulnerable for alcoholism and who would benefit from targeted prevention and intervention strategies. In addition, trait markers might assist clinicians in better diagnosing sub-populations of alcoholics who differ in their treatment needs. Furthermore, these markers could be an important step in research efforts to identify candidate genes (i.e., genes that are suspected to code for proteins that mediate alcohol’s actions on the brain and thus contribute to the development of alcoholism).

Researchers to date have proposed numerous potential trait markers of alcoholism. Although the validity of some of these markers could not be confirmed in subsequent experiments, other markers appear to be viable biological measures associated with the risk for alcoholism. This article highlights two such biochemical markers—platelet monoamine oxidase (MAO) and adenylyl cyclase (AC) activity levels—and emphasizes clinically relevant ways these trait markers might be used to help identify subgroups of alcoholics and to focus the search for potential candidate genes.

## What Is a Suitable Trait Marker?

The ideal trait marker for alcoholism would fulfill several requirements:

- It could be easily and reliably measured.
- It would be present only in people susceptible to developing the disorder—that is, it would be specific for alcoholism in general or for one of its subtypes.
- It would be either the product of a candidate gene or closely linked to one.
- It could be detected before the onset of alcoholism or during periods of stable abstinence.
- It would not be affected by other conditions, such as the use of other drugs or the presence of coexisting disorders.

For a common disease such as alcoholism, which is mediated via complex genetic and environmental interactions, however, it is not likely that one single marker exists. Previous genetic studies have shown that the familial transmission of alcoholism likely involves multiple genes and incomplete penetrance (i.e., not all persons inheriting the gene(s) will develop alcoholism) and that environmental factors also play a major role in its pathogenesis (Pardes et al. 1989). Because of this genetic heterogeneity (i.e., variability), researchers have focused on identifying more homogeneous subtypes of alcoholism and biological factors associated with these subtypes. (For more information on alcoholism subtypes, see sidebar, p. 178)

**Hypothesized Subtypes of Alcoholism**

Alcoholism is a heterogeneous disease, meaning that many different genetic and environmental factors cause or contribute to its development. To improve research into the causes of alcoholism and to develop new approaches to its prevention, diagnosis, and treatment, scientists try to categorize alcoholics into subgroups that share certain characteristics. A large number of different subtyping schemes exist. The three most commonly used schemes are described here.

***Primary Alcoholism Versus Primary ASPD With Secondary Alcoholism***

The distinction for subgrouping alcoholics into primary and secondary alcoholics is based on a person’s clinical history and the chronology of development of symptom clusters (Schuckit 1985). A person who develops alcohol dependence before the onset of any other major psychiatric disorder has primary alcoholism. People who exhibit a pattern of irresponsibility and of violating the rights of others (i.e., meet the criteria for antisocial personality disorder [ASPD]) before the onset of alcohol dependence are diagnosed with primary ASPD with secondary alcoholism.

***Type I Versus Type II Alcoholism***

Cloninger (1987) was the first researcher to describe type I and type II alcoholism subtypes. According to these classifications, type I, or “milieu-limited,” alcoholism occurs in both female and male offspring of alcoholic biological mothers and fathers and is influenced by postnatal environmental effects. This type of alcoholism usually is associated with adult onset and minimal criminality. Conversely, type II, or “male-limited,” alcoholism refers to an alcoholism subgroup that is transmitted primarily from alcoholic fathers to their sons and which often begins during adolescence (i.e., early onset alcoholism). This type of alcoholism rarely reacts to environmental factors and usually is associated with severe recurrent alcoholism and criminal behavior.

***Type A Versus Type B Alcoholism***

Babor and colleagues (1992) first described a subgrouping scheme for alcoholics that included type A and type B alcoholics, named after the Roman gods Apollo and Bacchus. Type A alcoholics are characterized by later onset, fewer childhood risk factors, less severe dependence, fewer alcohol-related problems, and less psycho-pathological dysfunction. Type B alcoholics typically exhibit an early onset of alcohol-related problems, higher levels of childhood risk factors and familial alcoholism, greater severity of dependence, multiple substance use, a long-term treatment history, greater psychopathological dysfunction, and greater life stress.

—*Robert M. Anthenelli and Boris Tabakoff*

ReferencesBaborTFHofmannMDelBocaFKHesselbrockVMMeyerREDolinskyZSRounsavilleBTypes of alcoholics I: Evidence for an empirically derived typology based on indicators of vulnerability and severityArchives of General Psychiatry495996081992163725010.1001/archpsyc.1992.01820080007002CloningerCRNeurogenetic adaptive mechanisms in alcoholismScience2364104161987288260410.1126/science.2882604SchuckitMAThe clinical implications of primary diagnostic groups among alcoholicsArchives of General Psychiatry42104310491985405168110.1001/archpsyc.1985.01790340021003

## Platelet MAO Activity

### Background and History

One of the most intensely studied proposed biological markers for a predisposition to some alcoholism subtypes is platelet MAO activity. MAO is an enzyme that is important for the breakdown of a variety of monoamine neurotransmitters^1^—a group of small molecules involved in the signal transmission between nerve cells—in the brain as well as in peripheral tissues. Monoamine neurotransmitters include dopamine, norepinephrine, and serotonin, all of which have been implicated in various phenomena related to the risk for developing alcoholism. These phenomena include a preference for consuming alcohol, the susceptibility to alcohol’s rewarding effects, the development of tolerance to alcohol’s effects, or personality characteristics (e.g., impulsivity) that predispose a person to repeated alcohol-related problems (Tabakoff and Hoffman 1992).

Mammals, including humans, have two types of MAO’s—MAO–A and MAO–B—that are the products of separate genes (Hsu et al. 1988). Furthermore, the two enzymes differ in their preference for the neurotransmitters they metabolize. Both MAO–A and MAO–B appear to break down dopamine efficiently (Donnelly and Murphy 1977). MAO–A, however, primarily metabolizes norepinephrine and serotonin, whereas MAO–B prefers biological substances, such as tyramine and phenylethylamine. Although MAO–A generally degrades serotonin more efficiently than MAO–B, the neurons (i.e., the brain cells) that use serotonin as a neuro-transmitter appear to contain primarily MAO–B (Westlund et al. 1985). Consequently, MAO–B may play an important role in metabolizing serotonin as well as other neurotransmitters in the brain. Certain blood cells (i.e., the platelets) also contain both serotonin and MAO–B (Donnelly and Murphy 1977). Platelets are easily accessible and MAO–B activity in the platelets, which probably correlates with brain MAO–B activity, is relatively simple to measure and genetically controlled (and thus heritable). Accordingly, scientists have studied platelet MAO extensively as a surrogate for brain MAO–B.^2^

Researchers first detected the potential association between low platelet MAO–B activity levels and alcoholism in the mid-1970’s (Fowler et al. 1982). Since then, many studies have demonstrated that alcoholics have lower platelet MAO activity levels than nonalcoholic sex-matched controls; however, these findings are not unanimous (Anthenelli et al. 1995). One explanation for the discrepant results is that alcoholism is clinically and genetically heterogeneous. In addition, early studies linked low platelet MAO activity not only to alcoholism but to a variety of other neuropsychiatric conditions (e.g., schizophrenia and mood disorders), leading to the hypothesis that low MAO activity may be a more general marker for the risk of developing certain forms of psychopathology (Buchsbaum et al. 1976). Other investigators have further expanded this hypothesis. For example, researchers have suggested that low MAO activity is associated with forms of “disinhibitory psychopathology,” including type II, or early onset, alcoholism and psychopathy^3^ (Oreland and Shaskan 1983).

### Heritability of Platelet MAO Activity in Alcoholic Families

Evidence that low platelet MAO activity is heritable in alcoholic families is based, in part, on empirical data but mainly rests on more indirect results. Several studies have demonstrated an association between a family history of alcoholism and reduced platelet MAO activity, whereas other studies have detected no such association (for a review, see Sher et al. 1994). Evidence supporting the heritability of low MAO activity levels primarily derives from studies of families with type II alcoholism. This alcoholism subtype is believed to be marked by an earlier age of onset of alcohol-related problems, more social and legal consquences of drinking, and a greater genetic predisposition for its development (Cloninger 1987).

### Platelet MAO and Early Onset Alcoholism

Several studies have indicated that low platelet MAO activity might be a marker for type II alcoholism (e.g., von Knorring et al. 1991). In these studies, male type II alcoholics exhibited lower platelet MAO activity levels than male type I alcoholics and nonalcoholic controls. The differences between type I and type II alcoholics were not consistent across several studies (Anthenelli et al. 1995; Yates et al. 1990). A recent analysis of data from the ongoing Collaborative Study on the Genetics of Alcoholism (COGA) demonstrated, however, that regardless of the subgrouping scheme employed (i.e., type I versus type II, type A versus type B, or primary versus secondary alcoholism), men with an earlier age at onset and a more severe course of alcohol-related problems had significantly lower platelet MAO levels than nonalcoholic men (Anthenelli et al. in press *a*). These data suggest that at least for men, decreased platelet MAO activity might serve as a marker for a subtype of alcoholism characterized by an earlier age at onset, a more pernicious course of problems related to alcohol and drug (AOD) use, and impulsive-aggressive personality characteristics. Results in women are less consistent, although most studies show that female alcoholics exhibit lower platelet MAO activity than nonalcoholic women (Lex et al. 1993).

### Other Factors Influencing Platelet MAO Activity

Many other traits and conditions, some of which are associated with alcoholism, also correlate with altered platelet MAO activity. Several intrinsic characteristics (e.g., gender, psychiatric and medical illnesses, metabolic factors, and personality traits) and extrinsic factors (e.g., cigarette smoking, other drug use, and differences in the methods used to obtain and analyze platelets) influence platelet MAO activity (Anthenelli et al. 1995; Fowler et al. 1982). For example, Anthenelli and colleagues (in press *a*,*b*) recently demonstrated in two unrelated samples of alcoholics that a broad diagnosis of alcohol dependence or even an early age of onset of alcoholism alone did not significantly predict platelet MAO activity levels when cigarette smoking, gender, and impulsive personality traits also were considered.^4^ These data provide further evidence that low platelet MAO activity may not be a specific marker for alcoholism but may be a more general indicator of a spectrum of disorders marked by disinhibition, impulsive aggression, and a predisposition for AOD abuse (Sher et al. 1994; Anthenelli et al. 1995).

### How Could Low Platelet MAO Activity Affect the Risk for Alcoholism?

As mentioned previously, alterations in MAO–B activity levels could potentially contribute to the predisposition for alcoholism in several ways (i.e., through alterations in the breakdown of the neuro-transmitters dopamine, norepinephrine, and serotonin). Although a discussion of each of these possible mechanisms is beyond the scope of this brief review article, the recent focus on serotonin’s role in the pathogenesis of early onset alcoholism and related disorders warrants further attention. Oreland and Shaskan (1983) hypothesized that low platelet MAO activity reflects low central nervous system (CNS) serotonin turnover because people with low platelet MAO activity levels have only low levels of serotonin’s degradation products in their cerebrospinal fluid. Furthermore, several indirect measures of serotonin function in the CNS frequently are reduced in people with early onset alcoholism and associated personality characteristics (e.g., impulsive and aggressive personality) (Virkkunen and Linnoila 1993). These observations suggest that low platelet MAO activity is a peripheral marker of altered serotonin-dependent neurotransmission in the CNS, which in turn could affect multiple factors that might contribute to the risk for alcoholism.

It may be premature, however, to correlate the brain’s serotonin activity only with MAO–B. A recent study of transgenic mice that completely lacked MAO–A demonstrated that depletion of MAO–A without affecting MAO–B also altered the levels of serotonin in the brain, of serotonin degradation products, and of aggression in the mice (Cases et al. 1995). Therefore, the final verdict on whether MAO–A or MAO–B is the primary enzyme involved in brain serotonin metabolism must await the results of further studies.

## Platelet AC Activity

### Background and History

AC is an enzyme used by cells, including the brain’s neurons, to relay signals from a cell’s exterior to its interior. Exterior signals in the form of neurotransmitters or hormones interact with specific proteins, or receptors, on the cell’s surface. Some of these receptors interact with another group of proteins, called guanine nucleotide-binding proteins (i.e., G proteins), in the cell membrane.^5^ G proteins, in turn, modulate AC activity. AC produces a substance called cyclic adenosine monophosphate (cAMP), which serves as a “second messenger” within the cell, for example, by activating other proteins or genes. A cell can receive both inhibitory and stimulatory exterior signals. An inhibitory G protein (G_i_) transmits inhibitory signals to AC, resulting in decreased cAMP production. Conversely, stimulatory signals are transmitted to AC via a stimulatory G protein (G_s_) and lead to increased cAMP production (Tabakoff and Hoffman 1992).

During the past decade, several researchers found that the AC activity levels in the platelets and in the lymphocytes (i.e., a type of white blood cell) of alcoholic subjects are lower than in the blood cells from nonalcoholic controls (Diamond et al. 1987; Tabakoff et al. 1988). Additional studies using the brain tissue of deceased alcoholics indicated that brain AC activity or the ability of brain AC to respond to stimulatory or inhibitory signals may be compromised in alcoholics (Ozawa et al. 1993). These findings have stimulated interest in the way that the intracellular regulatory pathways involving receptors, G proteins, AC, and cAMP might contribute to a person’s risk for developing alcohol dependence.

### Association of Platelet and Lymphocyte AC Activity With Alcoholism

In the initial study of platelet AC activity, the enzyme’s activity was lower in the samples of alcoholic subjects than in those of non-alcoholic control subjects, particularly when AC enzyme activity was stimulated through the stimulatory G protein, G_s_ (Tabakoff et al. 1988). These results held true even when the researchers controlled for factors such as age, race, smoking, and use of other drugs that could affect AC activity. Since then, scientists have repeated these results in smaller study populations in other geographical locations (Lex et al. 1993; Tabakoff and Hoffman 1989; Saito et al. 1994).

In the original investigation (Tabakoff et al. 1988), AC activity was low, even in alcoholics who reportedly had abstained from using alcohol for up to 4 years. These data suggested that the low AC activity in alcoholics might represent an inherent characteristic, a true trait marker reflecting a genetic predisposition to alcoholism, and did not result from chronic alcohol consumption. The hypothesis that platelet AC activity is an inherited trait in alcoholics and other people also is supported by recent findings (see below) that a single gene seems to be responsible for the inheritance of the level of platelet AC activity within families (Devor et al. 1991). Similar to certain other purported trait markers for alcoholism, such as a specific form of a receptor for the neurotransmitter dopamine (Cloninger 1991), however, the researchers speculate that low platelet AC activity may be associated with, but not genetically linked to, alcoholism^6^ (Devor et al. 1991). This finding indicates that the gene coding for AC may determine a person’s susceptibility to alcoholism rather than actually cause the disease (Greenberg 1993).

Researchers detected low AC activity not only in the platelets but also in the lymphocytes of alcoholics (Diamond et al. 1987). In addition, the alcoholics’ lymphocytes were more sensitive to alcohol than cells obtained from controls, even after extended culture in the laboratory (Nagy et al. 1988). These findings suggest that an inherent difference exists between the lymphocytes of alcoholics and those of control subjects. This difference, however, may not be attributable exclusively to variations in AC activity. Waltman and colleagues (1993) analyzed AC activity in lymphocytes from control subjects, “active” alcoholics, and alcoholics in a treatment program who had been abstinent for approximately 3 weeks. Compared with lymphocytes from the control subjects, the lymphocytes from the abstinent alcoholics showed decreased AC activities and exhibited three times higher levels of protein and messenger ribonucleic acid (mRNA) for an inhibitory G protein (G_i2α_). The AC activity in the active alcoholics did not differ from the control subjects. The investigators concluded that the increased level of G_i2α_ protein could account for the decreased AC activity in the cells of the abstinent alcoholics. The results further suggest that alcohol consumption may increase lymphocyte AC activity so that lower activity levels in the active alcoholics would be evident only after an extended period of abstinence.

Although these and other studies detected differences between alcoholics and control subjects in platelet and lymphocyte AC activity, the usefulness of AC activity as a marker for alcoholism remains somewhat controversial. For example, current analyses from the COGA project have found no differences in platelet AC activity between a large sample of alcoholics and nonalcoholic control subjects (T.-K. Li, personal communication; also see the section by Li in the article on COGA, pp. 234–235). The significance of this observation still must be determined, however, because the COGA sample was selected to emphasize particular characteristics of the alcoholic subjects. In this regard, it is interesting that the alcoholics in the COGA sample demonstrated low platelet MAO activity (Anthenelli et al. in press *a*). Previous studies that simultaneously assessed platelet MAO and AC activity also detected no correlation between these two markers (Tabakoff et al. 1988). It is possible, therefore, that low platelet MAO activity and low platelet AC activity define different alcoholic subtypes.

### Heritability of Platelet AC Activity in Alcoholism

Several studies have demonstrated the familial transmission of AC activity. One study indicated that one gene determined a large extent of G protein-stimulated AC activity, although several other genes also modestly affected AC activity (Devor et al. 1991). Lex and colleagues (1993) provided further support for the heritability of platelet AC activity with findings that nonalcoholic women with family histories of alcoholism exhibited lower basal and stimulated AC activities than women without such family histories. Similarly, the stimulated AC activity was lower in platelets of family history-positive alcoholic men than in platelets of family history-negative alcoholics or in non-alcoholic control subjects (Saito et al. 1994).

### Platelet AC and Early Onset Alcoholism

Studies investigating the correlation of platelet AC activity and early onset alcoholism still are relatively sparse. One recent study comparing type I and type II alcoholics found no differences between the groups in either basal or stimulated AC activity levels (Parsian in press). These results, coupled with studies demonstrating no significant correlation between platelet AC activity and platelet MAO activity (Tabakoff et al. 1988), indicate that platelet AC activity does not appear to be related to the risk for early onset alcoholism but might be associated with another subtype of alcoholism.

### How Could Low Platelet AC Affect the Risk For Alcoholism?

Preliminary studies indicate that intracellular mechanisms, including the AC/cAMP pathway, might be involved in producing tolerance to some of alcohol’s effects as well as in AOD effects related to reinforcement and craving (Nestler 1994). Although G protein-coupled receptors and AC activity exist in neurons throughout the brain and the peripheral tissues, these effects appear to be mediated mainly through a group of brain structures called the mesolimbic pathway. Growing evidence suggests that AOD’s primarily affect this pathway, which may be an important “reward circuit” in the brain that modulates drug reinforcement (i.e., the pleasurable drug effects) and craving.

The neurons of the mesolimbic pathway, which primarily secrete dopamine as a neuro-transmitter, originate in the brain stem and are connected to cells in the forebrain and particularly in the nucleus accumbens. The nucleus accumbens is an integrative area of the brain that receives input from several brain regions in addition to the brain stem. Through its integrative function, the nucleus accumbens plays an important role not only in mediating the perception of reward but also in learning and locomotor activity. AOD’s (e.g., cocaine and opiates) increase the dopamine-mediated neurotransmission in the nucleus accumbens. The increase may be partly responsible for the reinforcing effects of addictive drugs.

Dopamine transmits signals from one cell to the next by binding to certain proteins (i.e., receptors) on the signal-receiving neuron’s surface. These receptors then interact with other cellular components, such as the AC system. Researchers found that reinforcement requires the interaction of dopamine receptors with the AC system in the neurons whose cell bodies are located in the nucleus accumbens. For example, when cells in the nucleus accumbens were destroyed in laboratory animals, the animals reduced their self-administration of both heroin and cocaine (self-administration is an indicator of the reinforcing effects of these drugs) (Zito et al. 1985). Consequently, Nestler and colleagues (1993) suggested that the AC/cAMP-related signal transmission system in the nucleus accumbens might underlie reinforcement and craving for various drugs, including alcohol, cocaine, and heroin. Substantial additional research is needed, however, to clarify the role of brain AC in the reinforcing properties of alcohol and to relate platelet AC activity to phenomena occurring in the brain.

## Conclusion

The results of family, twin, and adoption studies provide evidence that genetic factors contribute to the development of alcoholism (Anthenelli and Schuckit 1992; National Institute on Alcohol Abuse and Alcoholism 1994). As with other common disorders associated with high rates of morbidity and mortality, alcohol researchers have sought to identify traits and genes that may increase the risk for the disorder. This article has reviewed the evidence that two biochemical markers, MAO activity and AC activity, may be associated with a predisposition to alcoholism in some people. These studies did not detect any genetic linkage between these two markers and alcoholism. It is possible, however, that the heterogeneity of alcoholism, as well as the tendency for assortative mating among alcoholics,^7^ may hamper any classical attempts to produce evidence of linkage between a genetic marker and the generic categorization of alcoholism. To circumvent some of these problems, future linkage analyses should categorize study subjects into particular alcoholism subtypes.

The search for markers of alcoholism is ongoing and extends beyond the two biochemical measures described here to include electrophysiological and genetic polymorphism markers discussed elsewhere in this journal issue. With these markers, researchers hope to achieve four main goals. First, biological markers of alcoholism might improve the diagnosis of alcohol dependence. Second, markers that can be detected before the onset of alcohol-related problems would allow researchers, physicians, and other health care providers to prevent the development of alcoholism through targeted intervention. Third, the markers could help elucidate the pathophysiology of the disorder. Finally, reliable markers might help guide treatment providers to develop better treatment strategies by either improving treatment matching techniques or by allowing intervention earlier in the disease process.

## Footnotes

1 For a definition of this and other technical terms used in this article, see central glossary, pp. 182–183.

2 Consequently, the term “platelet MAO” used throughout this article refers specifically to MAO–B.

3 The term “psychopathy” derives from the psychoanalytic literature and represents a personality type marked by dynamic features such as the inability to experience remorse or guilt and the manipulation of others to serve one’s own ends. Although frequently used interchangeably, “psychopathy” and “antisocial personality disorder” (ASPD) are not synonymous terms; only a subgroup of people diagnosed with ASPD according to the American Psychiatric Association’s *Diagnostic and Statistical Manual of Mental Disorders, Fourth Edition* exhibit high scores on the Psychopathy Checklist, Revised, developed by Hare (1991). In this article, psychopathy encompasses both ASPD and violent, impulsive criminal behavior.

4 Because evidence exists that exsmokers experience similar MAO activities as people who never smoked, it remains to be determined whether cigarette smoking alone explains most of the effects on platelet MAO activity in alcoholics (Anthenelli et al. in press *a;* Sher et al. 1994.)

5 For more information on G proteins, see the article by Miles, pp. 237–243.

6 This means that the gene(s) coding for low-stimulated AC levels may increase the risk for alcoholism rather than being a necessary or sufficient cause for its development.

7 Assortative mating means that a person with an alcoholic background is more likely than a nonalcoholic person to select an alcoholic partner. The mating of two people with a potential genetic predisposition for alcoholism increases the difficulty of tracing specific alcohol-related genetic markers in these people and their offspring.

## References

[b36-arhw-19-3-176] Babor TF, Hofmann M, DelBoca FK, Hesselbrock VM, Meyer RE, Dolinsky ZS, Rounsaville B (1992). Types of alcoholics I: Evidence for an empirically derived typology based on indicators of vulnerability and severity. Archives of General Psychiatry.

[b37-arhw-19-3-176] Cloninger CR (1987). Neurogenetic adaptive mechanisms in alcoholism. Science.

[b38-arhw-19-3-176] Schuckit MA (1985). The clinical implications of primary diagnostic groups among alcoholics. Archives of General Psychiatry.

